# Does benefits-of-breastfeeding language or risks-of-formula-feeding language promote more-positive attitudes toward breastfeeding among midwives and nurses?

**DOI:** 10.1186/s12884-023-05493-w

**Published:** 2023-03-11

**Authors:** Ayumi Toda, Keiko Nanishi, Akira Shibanuma

**Affiliations:** 1 Department of Reproductive Health Nursing, Graduate School of Health Care Sciences, Tokyo Medical and Dental University, 1-5-45 Yushima, Bunkyo-ku, Tokyo, Japan; 2 Office of International Academic Affairs, Graduate School of Medicine, the University of Tokyo, 7-3-1 Hongo, Bunkyo-ku, Tokyo, Japan; 3 Department of Community and Global Health, Graduate School of Medicine, the University of Tokyo, 7-3-1 Hongo, Bunkyo-ku, Tokyo, Japan

**Keywords:** Breastfeeding, Nurse, Education, Nursing, Midwife, Internet-based intervention

## Abstract

**Background:**

Midwives and nurses are crucial in breastfeeding support. Few studies have explored appropriate language for nursing education on breastfeeding. We assessed the impact of the language used on breastfeeding attitudes among midwives and nurses.

**Methods:**

A quasi-experimental study was conducted online in Japan among 174 midwives and nurses who had work experience in obstetrics or pediatrics. Participants were allocated to three groups to receive different text messages as the intervention (the benefit of breastfeeding for Group 1; the risk of formula feeding for Group 2; the importance of childcare for Group 3 as the comparison). The Japanese version of the Iowa Infant Feeding Attitude Scale (IIFAS-J) was used to assess breastfeeding attitudes before and after reading the texts. Also, participant reactions to the text were assessed by their responses to three statements. ANOVA, the chi-square test, and the t-test were used for outcome assessments.

**Results:**

The post-test IIFAS-J score was significantly higher than the pre-test score only for Group 1 (*p* <  0.01). The percentage of participants who agreed with the content of the text was 70.7% in Group 1 and 48.3% in Group 2. The percentage of participants who reported discomfort with the text was 34.5% in Group 1 and 55.2% in Group 2. No significant difference among groups existed regarding interest in the text. In all three groups, participants who agreed with the text had a higher post-test IIFAS-J score than those who disagreed with the text (6.85 points higher, *p* <  0.01 in Group 1; 7.19 points higher, *p* <  0.01 in Group 2; 8.00 points higher, *p* <  0.02 in Group 3). Discomfort with the text and interest in the text were associated with a significantly higher post-test IIFAS-J score in Group 1 and Group 2 but not in Group 3.

**Conclusions:**

“Benefits of breastfeeding” language, which conveys the information in a positive manner, appears to be more appropriate than “risks of infant formula” language for creating a positive attitude toward breastfeeding in nursing education.

**Trial registration:**

This study was registered in the University Hospital Medical Information Network Clinical Trials Registry (UMIN000023322). Registered 05/08/2016.

**Supplementary Information:**

The online version contains supplementary material available at 10.1186/s12884-023-05493-w.

## Introduction

Midwives and nurses play a crucial role in supporting breastfeeding [1–4], and their attitudes toward breastfeeding affect how they support breastfeeding. When midwives and nurses had neutral or negative attitudes toward breastfeeding, they often unnecessarily suggested using infant formula, leading to delayed initiation and premature cessation of breastfeeding [4]. On the other hand, mothers were more likely to initiate and continue breastfeeding when cared for by midwives and nurses with positive breastfeeding attitudes [1, 5]. Thus, midwives’ and nurses’ positive attitudes toward breastfeeding are essential in promoting breastfeeding.

Although the benefits of breastfeeding are often highlighted in health education, some health experts have criticized the presentation of breastfeeding as the intervention and formula feeding as the comparison. A systematic review of the presentation of infant feeding studies found that only 11% of abstracts named formula feeding as a health risk exposure. The authors argued that skew in the communication of research findings might reduce knowledge of and support for breastfeeding among health professionals [6]. McNiel et al. argued that presenting infant formula use as a control practice when reporting the benefits of breastfeeding implicitly defined formula feeding as a normative standard. Therefore, to promote exclusive breastfeeding as a standard, they chose to express evidence on the benefits of breastfeeding as risks of infant formula [7]. In line with this practice, Stuebe argued that when breastfeeding is promoted as “breast is best,” formula feeding is implicitly suggested as “good” or “normal” [8].

There is concern regarding how people interpret breastfeeding recommendations when the recommendations are based on the risks of infant formula to their babies’ health. A study of 434 university students in the United States found no significant difference in breastfeeding intention between those who received advocacy texts with “risks of formula” language, which presented the risks of formula feeding over breastfeeding, and those who received texts with “benefits of breastfeeding” language, which highlighted the benefits of breastfeeding over formula feeding. Furthermore, respondents who read the text with risk language were more likely to rate it as less trustworthy, less accurate, and less helpful than were those who read the text that stressed the benefits of breastfeeding. The authors concluded that use of risk language might not be advantageous for health promotion and might even be counterproductive to the goals of breastfeeding advocates [9]. Other researchers expressed concerns regarding a breastfeeding campaign that focused on the risks of formula, because it might elicit shame in women [10]. Thus, message content and tone should be carefully considered [11].

In Japan, “breast is best” has been a standard message from health organizations since the Ministry of Health and Welfare launched a breastfeeding promotion campaign in 1975 [12]. Nursing textbooks usually explain the benefits of breastfeeding rather than the risks of formula feeding [13]. Although the manner in which scientific evidence on infant feeding is presented to mothers and others remains a subject of discussion, little is known about appropriate language for nursing education. Therefore, this study compared the effects of “benefits of breastfeeding” language (i.e., providing infant feeding information with breastfeeding as the intervention and formula feeding as the comparison) and “risks of formula” language (i.e., providing the same information with formula feeding as the intervention and breastfeeding as the comparison) on attitudes toward breastfeeding among Japanese midwives and nurses with work experience in obstetrics and pediatrics. We further assessed how midwives and nurses reacted to the information. Three research questions addressed were as follows:

1. Do changes in breastfeeding attitudes differ between midwives and nurses who have read a “benefits of breastfeeding” text and those who have read a “risks of formula” text?
2. Which version of the text produces agreement with the text, comfort with the text, and interest in the text among midwives and nurses?
3. Are there actions to the text associated with attitudes toward breastfeeding after reading the text?

## Methods

### Design and participants

This quasi-experimental study was conducted online between August 10 and October 14, 2016. Participants were recruited through a study notification published on 1) a website managed by Medicus Shuppan Publishers Co., Ltd., a large Japanese publisher in nursing and medical sciences, 2) a job search site for midwives and nurses, and 3) Facebook. Macromill, Inc., an online survey company, posted the study invitation on the abovementioned media, and eligibility was confirmed by screening questions when the participant responded. The inclusion criteria were possession of a national nurse license and previous work experience in obstetrics or pediatrics, including neonatal intensive care units and growing care units. As of 2008, applicants for a midwife license in Japan must have a national nurse license.

Participants were allocated to one of three groups: Group 1 received a “benefits of breastfeeding” text, Group 2 received a “risks of formula feeding” text, and Group 3 received a control text. Participants answered the questionnaire before and after reading the intervention text. The group allocation was not random; instead, participants were allocated by a computer system that balanced the number of participants in each group. Specifically, a new participant was assigned to the group with the smallest number of participants who had completed the pre-test questionnaire. After completing the pre-test questionnaire, the text of the allocated group was presented to the participants, after which they were invited to a post-test questionnaire. The whole process took approximately 30 minutes. Although participants were encouraged to read the text before proceeding to the post-test questionnaire, skipping the reading was possible and not monitored.

### Blinding

As the intervention, participants were asked to read the text assigned to their group. Therefore, participants were not blinded to group allocation and could likely determine the allocation from the content of the text. The authors were blinded to the group allocation until the outcomes were analyzed and could not influence the responses.

### Texts

The texts for Group 1 and Group 2 were created in Japanese by using materials developed in English [9]. Wallace and Taylor developed two texts to convey the same information on infant feeding: one using benefit language and the other using risk language. For example, the message “breastfed children are less likely to suffer from infectious illnesses and their symptoms” in the benefits text was converted to “formula-fed children are more likely to suffer from infectious illnesses and their symptoms” in the risks text. The present authors created the Japanese version of these texts with the permission of Wallace and Taylor, and the back-translation method was used for the translation. Specifically, the first author translated the original English version into Japanese, and a Japanese–English bilingual graduate student of public health who was blinded to the original version back-translated it into English. The authors compared the back-translated version and original English version. In the event of a discrepancy in meaning, the first author revised the Japanese translation. The process was repeated until the authors agreed that the Japanese translation was semantically equivalent to the original.

Before translation, the authors checked the relevance of the content of the texts. First, the authors assessed if the textual information was consistent with existing evidence in 2016. Systematic reviews and a policy statement published after the development of the original English version were examined [14–21]. No corrections were made to the texts, as all statements were consistent with evidence discussed in the reviews. After the Japanese translation was completed, the content of the texts was reviewed by a pediatrician and a breastfeeding researcher in Japan who were not part of the research team. They confirmed that the texts were relevant for midwives and nurses in Japan. The final versions of the back-translated texts are shown in Supplementary Material 1.

The control group (i.e., Group 3) received a brochure on healthy pregnancy and delivery developed by the Ministry of Health, Labour and Welfare [22]. The brochure was published online on the Ministry’s website for free use. The authors chose it as a control text because it did not mention infant feeding and was about the same length as the intervention texts.

### Measurement

#### Attitude toward breastfeeding

Attitude toward breastfeeding was measured twice in the pre-test and post-test questionnaires by the Iowa Infant Feeding Scale-Japanese version (IIFAS-J) [23]—a Japanese version of the Iowa Infant Feeding Attitude Scale developed by de la Mora and Russell in the United States [24]. The IIFAS-J was tested for reliability and validity among 673 mothers, and one item was omitted from the original 17-item version [23]. The IIFAS-J thus consists of 16 items rated on a 5-point Likert-type scale (1 = strongly disagree to 5 = strongly agree). The total IIFAS-J score ranges from 16 to 80; lower scores indicate a more positive attitude toward formula feeding, while higher scores indicate a more positive attitude toward breastfeeding [23]. In this study, the Cronbach alpha values for pre-test and post-test surveys were 0.78 and 0.80, respectively, indicating that the scale was sufficiently reliable.

#### Nurse reactions to the texts

The reaction of participants to the texts was assessed by their response to the three statements in the post-test questionnaire: “I can agree with the content of the text,” “The text makes me uncomfortable,” and “I’m interested in the text.” For all items, participants were asked to respond by using a 5-point Likert-type scale, and responses were dichotomized after checking the distribution of answers (Supplementary Table 1). The responses “strongly agree” and “agree” to the first statement were categorized as agreement with the text; all other responses were categorized as disagreement with the text. The responses “strongly agree,” “agree,” and “neither” to the second statement were categorized as comfort with the text; all other responses were categorized as discomfort with the text. The responses “strongly agree” and “agree” to the last statement were categorized as interest in the text; all other responses were categorized as lack of interest in the text.

### Background characteristics of participants

The pre-test questionnaire assessed the sociodemographic and employment characteristics of the participants. In addition to age, gender, total number of years of work experience as a nurse, possession of an advanced license in addition to the nursing license, education level, having a child (children), and recognition of the benefits of breastfeeding and risks of formula feeding were measured [25–27].

### Sample size estimation

A previous study of pregnant women reported a mean IIFAS-J score of 62 for those who intended to breastfeed exclusively. We aimed to increase the IIFAS-J score by 3 points by using benefit language to present the latest infant feeding information. We expected that use of risk language to present the same information would not have an equivalent effect. Specifically, we assumed that the control group would have a mean score of 62 at both the pre-test and post-test and designed the intervention for Group 1 to increase the mean score from 62 to 65 (SD 5). To detect a difference in pre-test score between Group 1 and the control with 80% power and a two-sided alpha of 5%, we estimated that a minimum sample size of 44 for each group would be necessary (http://clincalc.com/Stats/SampleSize.aspx). To account for the possibility of drop-outs, we enrolled a total of 147 participants.

### Data analysis

Four assumptions were tested: 1) the texts using benefit language and risk language would increase IIFAS-J scores, 2) the improvement in IIFAS-J score would be greater for Group 1 (i.e., those who read the benefits text) than for the other groups, 3) benefit language would be received more favorably than risk language, and 4) participants who reacted favorably to a text would have a more positive attitude toward breastfeeding in post-test measurement than did those who read the risks text. Analysis of Variance (ANOVA) or the chi-square test of independence was used to assess between-group differences in nurse characteristics. The paired t-test was used to compare mean differences in pre-test and post-test IIFAS-J scores within each group. ANOVA was used to compare mean IIFAS-J scores among groups at baseline and after the intervention, and change in IIFAS-J score after the intervention. The chi-square test of independence was used to compare how participants in the three groups reacted to the texts. The t-test was used to assess the association between receiving the text favorably and post-test IIFAS-J scores. Stata version 13.1 was used to analyze all the data (Stata Corp. LLC, College Station, Texas, USA), and a *p*-value less than 0.05 was considered to indicate statistical significance.

## Findings

### Participant flow

**Fig. 1:**
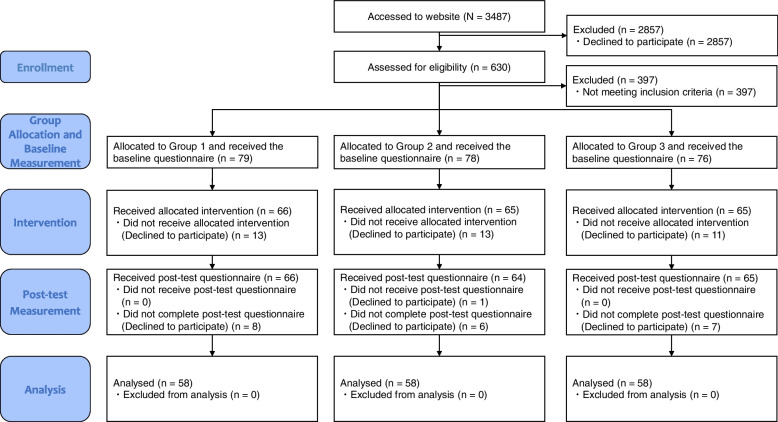
Participant flow diagram

Figure 1 shows the participant flow diagram. Of the 3487 individuals who accessed the study’s website, 630 proceeded to receive detailed information about study participation and responded to the screening questions. Among them, 397 did not meet the inclusion criteria and were not invited to the study. Consequently, the 233 midwives and nurses considered eligible were allocated to one of the three groups after informed consent was obtained. Ultimately, 174 participants completed both the pre-test and post-test questionnaires and were included in the analysis.

### Characteristics of participants

Table 1 shows the characteristics of participants by group (that is, the allocation of interventions). Among the groups, the characteristics of the participants were not significantly different, except for experience in the workplace. More participants in Group 1 had experience working in obstetric wards, as compared with Group 2 and Group 3 (i.e., the control) (*p* = 0.02). More participants in Group 1 had experience working in pediatric wards (*p* <  0.01) and neonatal intensive care units/growing care units (*p* <  0.01), as compared with the other groups. All 174 participants reported hearing of the benefits of breastfeeding, and majority reported hearing of the risks of formula feeding.

**Table 1:** Characteristics of participants

	Group 1^**a**^ (***n*** = 58)		Group 2^**b**^ (***n*** = 58)		Group 3^**c**^ (***n*** = 58)		***P***-value^**d**^
	***n (%)***	***Mean [SD]***	***n (%)***	***Mean [SD]***	***n (%)***	***Mean [SD]***	
**Age**		35.6 [7.5]		34.9 [7.2]		34.3 [6.6]	0.66
**Total number of years of work experience as a nurse**							
		12.0 [7.6]		11.1 [6.9]		10.0 [6.3]	0.32
**Possession of advanced license in addition to nursing license**							
Midwife license	32 (55.2)		33 (56.9)		29 (50.0)		0.74
Public Health Nurse license	30 (51.7)		31 (53.4)		24 (41.4)		0.37
**Educational experience**							0.76
University/Master’s degree	34 (58.6)		32 (55.2)		30 (51.7)		
3-year curriculum	24 (41.4)		26 (44.8)		28 (48.3)		
**Have a child/children?**							0.31
Yes	39 (67.2)		32 (55.2)		32 (55.2)		
**Heard of breastfeeding benefits?**							
Yes	58 (100.0)		58 (100.0)		58 (100.0)		
**Heard of infant formula risks?**							0.67
Yes	51 (87.9)		48 (82.8)		48 (82.8)		

^a^Group 1 received a text highlighting the benefits of breastfeeding

^b^Group 2 received a text highlighting the risks of formula feeding

^c^Group 3 received a control text

^d^Analysis of variance for continuous variables; chi-square test for categorical variables

### Effect of texts on attitudes toward breastfeeding

The paired t-test showed that the mean (SD) IIFAS-J score improved significantly, from 63.5 (7.3) to 65.3 (8.0), in Group 1 (the difference between the pre-test and post-test was 1.8 points, *p* < 0.01). In contrast, the IIFAS-J scores did not improve in Group 2 or Group 3. ANOVA showed that change in mean IIFAS-J score after reading the text did not significantly differ among the three groups (*p* = 0.21) (Table 2).

**Table 2:** Pre-test and post-test IIFAS-J scores

	Group 1^**a**^ (***n*** = 58)	Group 2^**b**^ (***n*** = 58)	Group 3^**c**^ (***n*** = 58)	***P***-value^**d**^
	***Mean [SD]***	***Mean [SD]***	***Mean [SD]***	
**Pre-test**	63.5 [7.3]	64.4 [7.5]	63.3 [8.7]	0.70
**Post-test**	65.3 [8.0]	65.1 [7.8]	64.1 [7.8]	0.66
**Difference**	1.8 [3.9]	0.7 [3.8]	0.8 [3.9]	0.21
***P*-value**^**e**^	< 0.01	0.16	0.13	

^a^Group 1 received a “benefits of breastfeeding” text

^b^Group 2 received a “risks of infant feeding” text

^c^Group 3 received a control text

^d^*P*-value for analysis of variance

^e^*P*-value for paired *t*-test within group

### Participants’ reactions to the texts

The texts elicited different reactions among the participants (Table 3). While 70.7% of participants who received the benefits text (i.e., Group 1) agreed with the text, only 48.3% of those who received the risks text (i.e., Group 2) agreed with the text. Similarly, while only 34.5% of participants who received the benefits text (i.e., Group 1) reported discomfort with the text, 55.2% of those who received the same information in the risks text (i.e., Group 2) reported discomfort with the text. In Group 1, 79.3% of participants reported that the text was interesting, while 74.1% of the participants in Group 2 reported that the text was interesting. In the control group (i.e., those receiving a text unrelated to infant feeding), the vast majority reported that they agreed with the text, some reported discomfort, and most found it interesting. When the three groups were compared, there were significant differences in agreement (*p* < 0.01) and discomfort (*p* < 0.01) with the text, but no significant difference in interest in the text.

**Table 3:** Reaction of participants to the study texts

	Group 1^**a**^ (***n*** = 58)	Group 2^**b**^ (***n*** = 58)	Group 3^**c**^ (***n*** = 58)	***P***-value^**d**^
	***n (%)***	***n (%)***	***n (%)***	
				< 0.01
Agreement with text	41 (70.7)	28 (48.3)	53 (91.4)	
				< 0.01
Uncomfortable with text	20 (34.5)	32 (55.2)	8 (13.8)	
				0.56
Interested in text	46 (79.3)	43 (74.1)	41 (70.7)	

^a^Group 1 received a text highlighting the benefits of breastfeeding

^b^Group 2 received a text highlighting the risks of formula feeding

^c^Group 3 received a control text

^d^Chi-square test

### Difference in post-test IIFAS-J scores in relation to reaction to the text

**Fig. 2:**
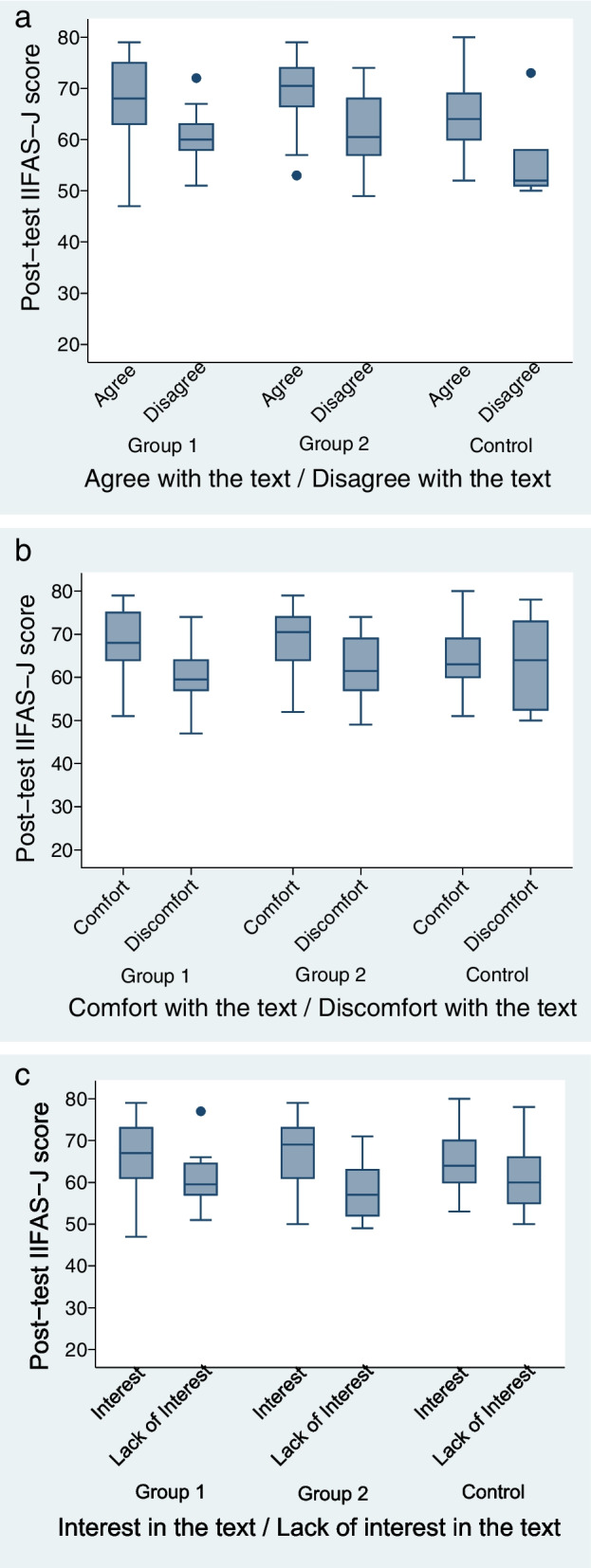
Comparison of post-test IIFAS-J scores between those who received the text favorably and those who did not within each group. **a** Mean scores of post-test IIFAS-J scores (compared those who agreed with the text and those who disagreed with the text) **b** Mean scores of post-test IIFAS-J scores (compared those who reported discomfort with the text and those who did not) **c** Mean scores of post-test IIFAS-J scores (compared those who were interested in the text and those who lacked interest)

Differences in post-test IIFAS-J scores were assessed within each group in relation to agreement with (Fig. 2a), discomfort with (Fig. 2b), and interest in (Fig. 2c) the text. In all three groups, participants who agreed with the text had higher post-test scores (Fig. 2a). Specifically, in Group 1, the post-test score was 6.85 points higher among participants who agreed with the text than among those who did not (*p* < 0.01). Similarly, in Group 2, the post-test score was 7.19 points higher among participants who agreed with the text than among those who did not (*p* < 0.01). In Group 3, the mean post-test score was higher among participants who agreed with the text than among those who did not (8.00 points, *p* < 0.02). Discomfort with the text was associated with lower post-test scores in Group 1 and Group 2 (Fig. 2b). In Group 1, the mean post-test score was 7.35 points lower for participants who reported discomfort with the text than for those who did not (*p* < 0.01). Likewise, in Group 2, the mean post-test score was 6.86 points lower for participants who reported discomfort with the text than for those who did not (*p* < 0.01). In Group 3, discomfort with the text was not significantly associated with post-test score (*p* = 0.79). Finally, interest in the text was associated with higher post-test IIFAS-J scores in Group 1 and Group 2 (Fig. 2c). In Group 1, the mean post-test score was 5.43 points higher for participants who were interested in the text than for those who were not (*p* < 0.05). In Group 2, the mean post-test score was 9.80 points higher for participants who were interested in the text than for those who were not (*p* < 0.01). In Group 3, interest in the text was not significantly associated with post-test score (*p* = 0.15).

## Discussion

The present findings suggest that “benefits of breastfeeding” language is better than “risks of infant formula” language for producing a positive attitude to breastfeeding in nursing education. Only the benefits text significantly improved the attitude to breastfeeding. Second, the benefits text was received more favorably than the risks text. Specifically, most participants who received the benefits text agreed with it, while most participants who received the risks text found it uncomfortable. Moreover, agreement and lack of discomfort with a text were associated with more-positive attitudes toward breastfeeding after reading it.

Comparison of pre-test and post-test IIFAS-J scores indicated that only the benefits text significantly improved midwives’ and nurses’ attitudes toward breastfeeding; the same information presented with risk language was not effective. This finding was consistent with a study of undergraduate students [9]. The authors rejected the assumption that risk language advocacy would be more effective in promoting breastfeeding intention and suggested that such language might create a backlash against its negative tone. Our study findings suggest that their ideas might be applicable to midwives and nurses with work experience in obstetrics, neonatology, and pediatrics. We found that risk language did not enhance midwives’ and nurses’ attitudes toward breastfeeding. Risk language was not only ineffective, it was received less favorably than benefit language. Midwives and nurses were less likely to agree and more likely to feel discomfort when infant feeding information was presented in risk language.

When change in IIFAS-J score was compared among the three groups, neither of the intervention groups (i.e., those receiving the benefits text and the risks text) showed more improvement than the group receiving the control text. Most of the participants reported that they had heard of the benefits of breastfeeding and the risks of infant formula before participating in the study. Nurses who are knowledgeable about breastfeeding often have a positive attitude toward breastfeeding [28]. The mean pre-test IIFAS-J scores of all the present groups were higher than the mean IIFAS-J score, 61.0, in a previous study of 781 pregnant women in their third trimester [23]. Therefore, midwives’ and nurses’ preliminary knowledge of infant feeding and their already positive attitude to breastfeeding might have obscured the effects of the present intervention texts.

Participants who favorably received the infant feeding text (i.e., were in agreement and felt no discomfort) had higher post-test IIFAS-J scores than did those who felt less favorably about the text in both intervention groups. This finding suggests that the reader’s feelings might impact the effectiveness of the intervention. Participants received the information more favorably when it was presented as the benefits of breastfeeding; thus, presenting infant feeding information that focuses on the benefits of breastfeeding may be more effective for promoting breastfeeding support among midwives and nurses. In addition, when any information is presented to midwives and nurses as a risk of infant formula use, it should be presented in a way that is agreeable and does not cause discomfort.

When interpreting our results, it should be noted that our study evaluated the language and not the content of the texts. Nursing education on breastfeeding must encompass more than the benefits of breastfeeding. Lactating mothers expect health professionals to provide sensitive responses to their emotional needs, in addition to relevant knowledge and practical advice [29, 30]. Thus, nursing education to support breastfeeding should encompass a wide range of supporting techniques, such as counselling skills that encourage empathy when working with lactating mothers [31]. Our study findings suggest that participants’ feelings about educational material, and not simply the information itself, might affect the effectiveness of such material. Future studies should explore how to best present infant feeding information to midwives and nurses as part of a comprehensive curriculum that encompasses all the necessary skills for breastfeeding support.

There are several other limitations of the study. The present findings might also be affected by selection bias because of the nature of online surveys [32] and the low response rate. As is the case for many online surveys, we know nothing about participant characteristics beyond the background data we collected. Therefore, the participants may have had characteristics that differed from those of midwives and obstetric and pediatric nurses in Japan. Also, participants were likely to be frequent users of internet services and social media and to be interested in infant feeding support. Next, participants were not forced to read the intervention texts; therefore, some participants might have skipped reading the intervention materials, which could have reduced the effectiveness of the intervention. Finally, the current study was conducted more than 6 years ago. After the data were collected, Japan’s Ministry of Health, Labor and Welfare revised its guidelines for health professionals on the feeding of infants and young children [33] and removed the term “breastfeeding promotion” from the text, in consideration of the feelings of mothers who choose to formula feed. Therefore, midwives and nurses may be more sensitive in using risk language than when we conducted the study.

Notwithstanding these limitations, the current study has value in assessing the language used in nursing education. We conclude that a text that presented the benefits of breastfeeding improved breastfeeding attitudes and that most midwives and nurses received it favorably. We found no advantage in using risk language to present infant feeding information. Future studies should attempt to identify the most appropriate way to convey infant feeding information as part of comprehensive nursing education to improve breastfeeding support.

## Conclusions

Only the benefits text significantly improved the attitude of midwives and nurses to breastfeeding. In addition, the benefits text was received more favorably than the risks text. Moreover, agreement and lack of discomfort with a text were associated with more-positive attitudes toward breastfeeding after reading it. Therefore, “benefits of breastfeeding” language, which conveys the information in a positive manner, appears to be more appropriate than “risks of infant formula” language for producing a positive attitude to breastfeeding in nursing education. If information is to be presented as a risk of formula feeding, it should be delivered in a way that is agreeable and less likely to cause discomfort.

## Supplementary Information

**Additional file 1.**

**Additional file 2: Supplementary Table 1.** Responses to items regarding participant reactions to the texts.

## Data Availability

Data used or analyzed in this study are available from the corresponding author on reasonable request.

## Abbreviations

IIFAS-J: Japanese version of Iowa Infant Feeding Attitude Scale
ANOVA: Analysis of Variance
SD: Standard Deviation
