# Supplementary material for: Does benefits-of-breastfeeding language or risks-of-formula-feeding language promote more-positive attitudes toward breastfeeding among midwives and nurses?
Source: BMC Pregnancy Childbirth. 2023 Mar 11;23:163. doi: 10.1186/s12884-023-05493-w (PMC10007738; doi:10.1186/s12884-023-05493-w)
Supplement: Supplementary file 1 — Additional file 1. [file 12884_2023_5493_MOESM1_ESM.docx]

**Supplementary Material 1.**

**Benefits of breastfeeding**

According to the World Health Organization, human milk is the best way to nourish infants.

Human milk is beneficial not only to the child but also to the mother and society.

The child can fulfill his or her full growth potential when raised with human milk and will also be protected from acute and chronic diseases.

Human milk is good for the child not only after birth, but the benefits can also be seen many years after breastfeeding stops.

When compared to a child raised with formula milk, a child raised with human milk is healthier, suffers less, and has the disease for a shorter period of time.

Research has found the following benefits.

When compared to a child raised with formula milk, a child raised with human milk has/is:

- Higher scores in cognitive and IQ tests. The same goes for a visual acuity test.
- Lower risk of SIDS.
- Less likely to suffer from infectious diseases and the associated symptoms (diarrhea, otitis media, respiratory infection, meningitis).
- Less susceptible to Crohn’s disease.
- When given human milk for 6 months or more after birth, the child is less susceptible to Hodgkin’s lymphoma.
- Less susceptible to early onset diabetes if the child has a family history of diabetes when given human milk exclusively at least 3-4 months after birth.
- Less susceptible to asthma and eczema.
- Less susceptible to obesity.
- Less susceptible to tooth decay and chewing abnormality.

**NOTE** *This text was adapted from Wallace and Taylor and modified by the researcher based on the latest information from the World Health Organization and the American Academy of Pediatrics and the latest systematic reviews. This is an English translation of the original material provided to participants in Japanese and should be used for informational purposes only. It has not been tested in English for use among English-speaking mothers*.

**Risks of infant formula**

According to the World Health Organization, formula milk is not the best way to nourish infants.

Formula milk has many disadvantages, not only to the child, but also to the mother and the society.

When you feed a child with formula milk, the child cannot fulfil his or her full growth potential, in addition to being exposed to risks for acute and chronic diseases.

A child will be disadvantaged when not raised with human milk not only after birth, but the effect will last for many years even after the child stops drinking formula milk.

When compared to a child raised with human milk, a child raised with formula milk is more susceptible to illness, suffers more, and has longer duration of the illness.

Research has found the following risks of formula milk.

When compared to a child raised with human milk, a child raised with formula milk has/is:

- Lower scores in cognitive ability and IQ tests. The same goes for a visual acuity test.
- Higher incidence of SIDS.
- More likely to suffer from infectious diseases and the associated symptoms (diarrhea, otitis media, respiratory infection, meningitis).
- More susceptible to Crohn’s disease.
- When not given human milk for 6 months or more after birth, the child is more susceptible to Hodgkin’s lymphoma.
- More susceptible to early onset diabetes if the child has a family history of diabetes when given formula milk at least 3-4 months after birth.
- More susceptible to asthma and eczema.
- More susceptible to obesity.
- More susceptible to tooth decay and chewing abnormality.

**NOTE** *This text was adapted from Wallace and Taylor and modified by the researcher based on the latest information from the World Health Organization and the American Academy of Pediatrics and the latest systematic reviews. This is an English translation of the original material provided to participants in Japanese and should be used for informational purposes only. It has not been tested in English for use among English-speaking mothers*.
