# Supplementary material for: Does benefits-of-breastfeeding language or risks-of-formula-feeding language promote more-positive attitudes toward breastfeeding among midwives and nurses?
Source: BMC Pregnancy Childbirth. 2023 Mar 11;23:163. doi: 10.1186/s12884-023-05493-w (PMC10007738; doi:10.1186/s12884-023-05493-w)
Supplement: Supplementary file 2 — Additional file 2: Supplementary Table 1. Responses to items regarding participant reactions to the texts. [file 12884_2023_5493_MOESM2_ESM.docx]

Supplementary Table 1. Responses to items regarding participant reactions to the texts

| **I can agree with the content of the text.** | | | | | |
| --- | --- | --- | --- | --- | --- |
|  | 1 : Strongly disagree | 2 : Disagree | 3 : Neither disagree or agree | 4 : Agree | 5 : Strongly agree |
| Group 1 | 0 | 2 | 15 | 17 | 24 |
| Group 2 | 3 | 8 | 19 | 19 | 9 |
| Group 3 | 0 | 0 | 5 | 25 | 28 |
| Total | 3 | 10 | 39 | 61 | 61 |
| **The text makes me uncomfortable.** | | | | | |
|  | 1 : Strongly disagree | 2 : Disagree | 3 : Neither disagree or agree | 4 : Agree | 5 : Strongly agree |
| Group 1 | 20 | 18 | 12 | 6 | 2 |
| Group 2 | 16 | 10 | 15 | 12 | 5 |
| Group 3 | 32 | 18 | 8 | 0 | 0 |
| Total | 68 | 46 | 35 | 18 | 7 |
| **I'm interested in the text.** | |  |  |  |  |
|  | 1 : Strongly disagree | 2 : Disagree | 3 : Neither disagree or agree | 4 : Agree | 5 : Strongly agree |
| Group 1 | 1 | 0 | 11 | 24 | 22 |
| Group 2 | 2 | 4 | 9 | 18 | 25 |
| Group 3 | 0 | 2 | 15 | 27 | 14 |
| Total | 3 | 6 | 35 | 69 | 61 |
